# Clinical Insights Into a Rare Left Main Coronary Artery Aneurysm

**DOI:** 10.7759/cureus.86328

**Published:** 2025-06-18

**Authors:** Boone Singtong, Kush Kapadia, Chris Aboujaoude, Swatam Jain, Yi McWhorter

**Affiliations:** 1 Internal Medicine, HCA Mountain View Hospital, Las Vegas, USA; 2 Internal Medicine, HCA Healthcare: MountainView Hospital, Las Vegas, USA

**Keywords:** cardiothoracic intensive care, cardio vascular disease, giant coronary artery aneurysm, left main coronary artery aneurysm, non obstructive coronary artery disease

## Abstract

This case report presents a 61-year-old female with hypertension and hyperlipidemia who developed giant coronary artery aneurysms (GCAA). Coronary artery aneurysms (CAA) are rare, with GCAA being even rarer, occurring in a very small percentage of CAA cases. The etiology of GCAA remains unclear. While CAAs are often asymptomatic and discovered incidentally, they can lead to severe complications such as myocardial infarction, sudden cardiac death, or rupture. Imaging revealed a significant aneurysm in the coronary bifurcation - initially, the patient presented with atypical chest pain. Patient underwent atypical chest pain workup, including stress test, which was negative. Given the patient’s age and comorbidities, the patient underwent a CT angiogram, which revealed a significant aneurysm at the coronary bifurcation into the left anterior descending (LAD) and circumflex arteries. This has been followed over a number of years with serial CT angiograms. A recent CT angiogram reveals a further increase in size of the aneurysm, almost approaching 1 cm. The patient underwent elective coronary angiography, revealing 30% stenosis in the LAD artery and other abnormalities. Given the growing size of the coronary aneurysm, the decision was made to perform surgical repair, including coronary artery bypass grafting. The patient was stabilized in the intensive care unit and was subsequently discharged.

## Introduction

Coronary artery aneurysms (CAA) are uncommon findings in the adult population, typically identified incidentally during coronary imaging. Even more infrequent are giant coronary artery aneurysms (GCAA), which represent a particularly rare subset of CAA, occurring in approximately 0.02%-0.2% of all CAA cases. Clinical presentation can vary from asymptomatic to symptomatic, including acute coronary syndrome, angina pectoris, or dyspnea. Sometimes it can mimic a mediastinal mass or cardiac tumor. The gold standard to diagnose CAA is coronary angiography, which provides the aneurysm’s anatomy [1]. A CAA is generally defined as a localized dilation of a coronary artery segment exceeding 50% of the diameter of the adjacent normal segment, with a prevalence on diagnostic coronary angiography ranging from 1.4% to 4.9%, depending on the patient population and diagnostic criteria used [2,3].

A CAA is generally defined as a localized dilation of a coronary artery segment exceeding 50% of the diameter of the adjacent normal segment [2]. The prevalence of CAAs identified on diagnostic coronary angiography ranges from 1.4% to 4.9%, depending on the patient population and diagnostic criteria used [3].

Despite advances in cardiovascular imaging and management, the etiology of GCAA remains poorly understood. One theory behind this is that the matrix metalloproteinases (MMPs) cause increased proteolysis in extracellular matrix proteins, resulting in the development of coronary aneurysms. In adults, atherosclerosis is the most frequently implicated cause, accounting for the majority of CAA cases. In contrast, CAAs identified in pediatric and the young adult population are more commonly associated with vasculitides such as Kawasaki disease and Takayasu arteritis. Other less common causes may include connective tissue disorders, congenital anomalies, trauma, infections, or iatrogenic factors following percutaneous coronary interventions. The development of CAA is still unclear [4].

We report the case of a 61-year-old female with a medical history of hypertension and hyperlipidemia who was found to have a previously undiagnosed GCAA, contributing to the limited but growing body of literature on this rare cardiovascular entity.

## Case presentation

**Figure 1:**
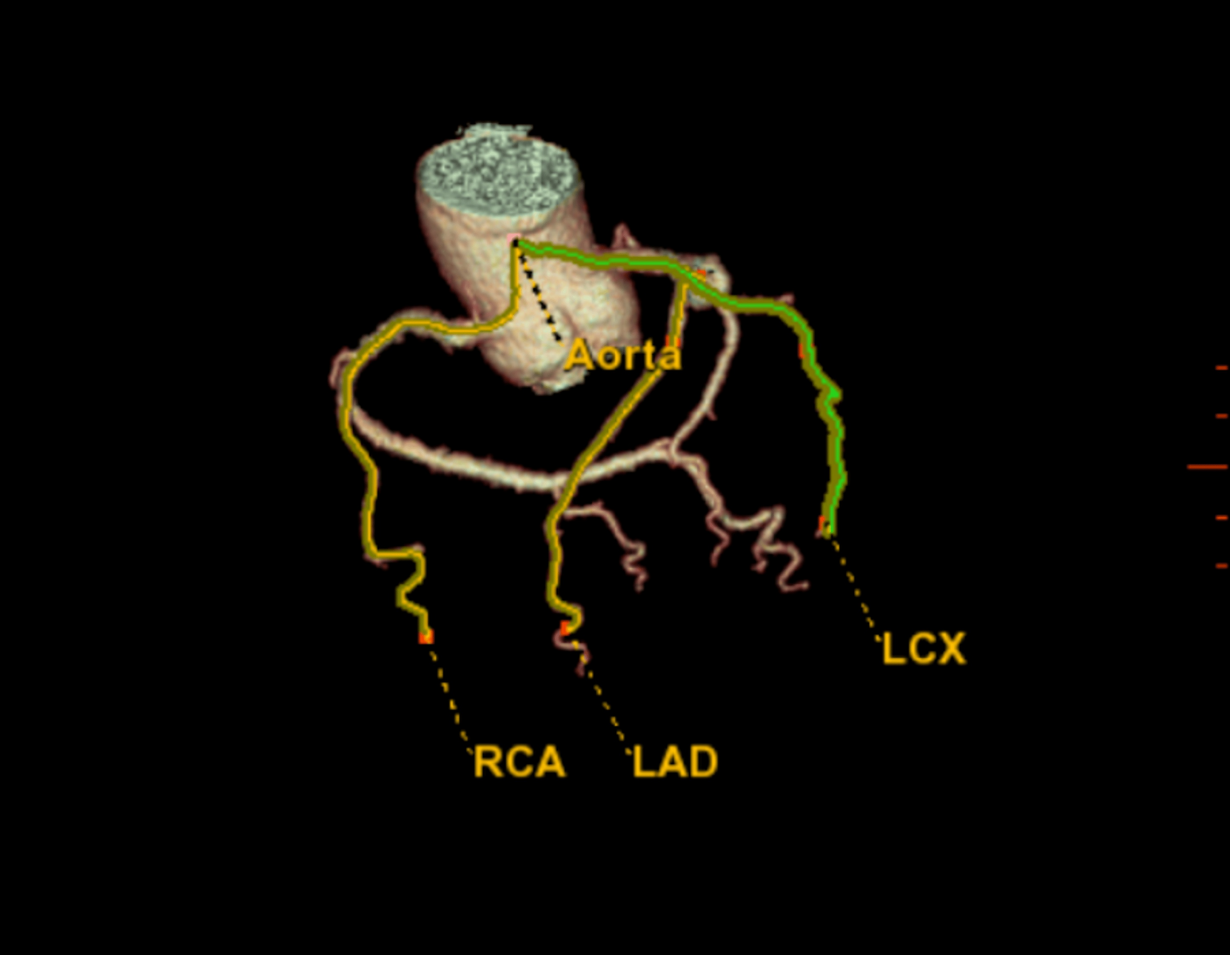
Diagram of 3D CTA coronary RCA: right coronary artery; LAD: left anterior descending artery; LCX: left circumflex artery; CTA: computed tomography angiography

**Figure 2:**
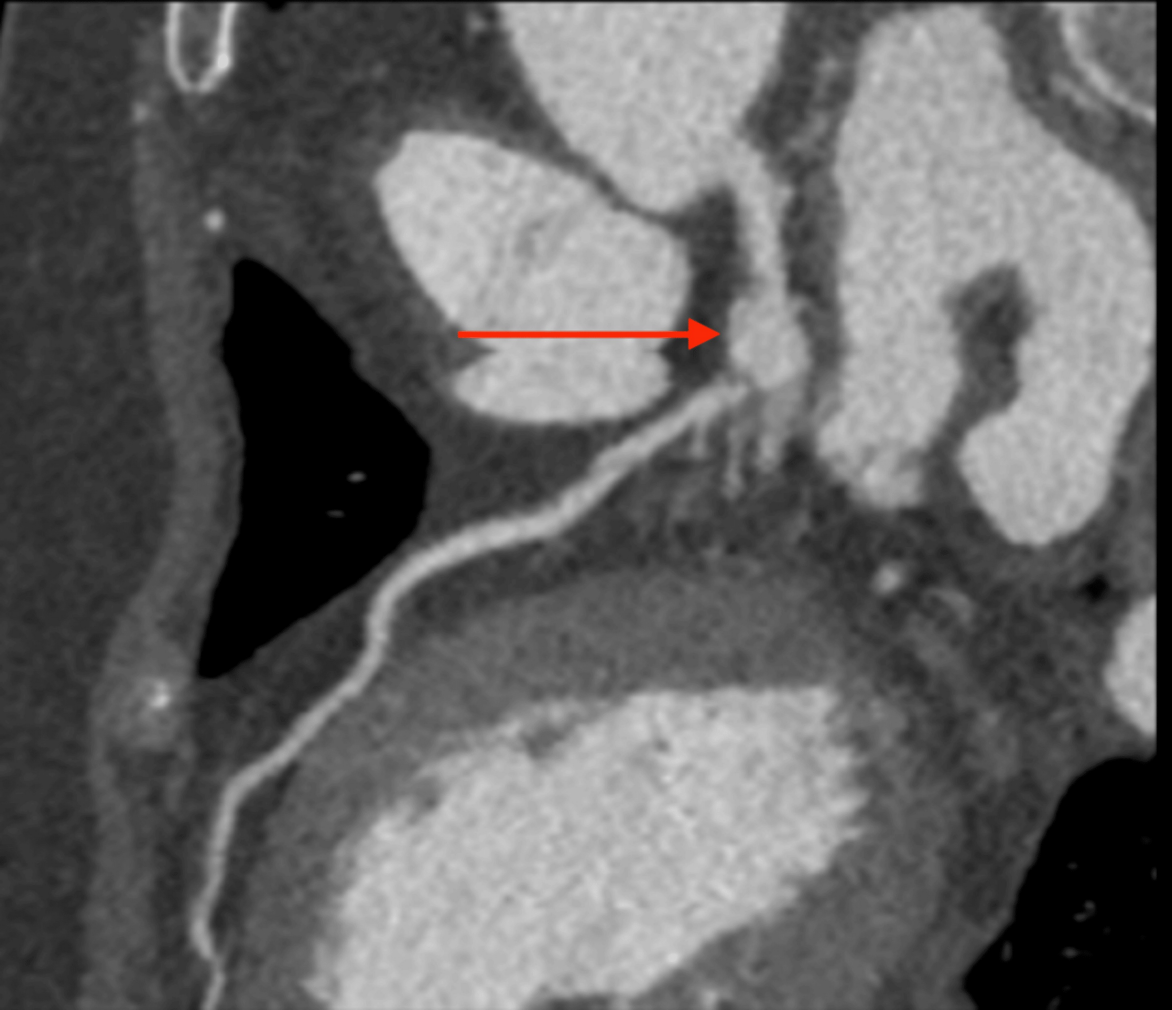
Computed tomographic angiogram of coronary arteries showed an 11 mm long by 9 mm wide nonthrombosed aneurysm emanating out of the distal end of the left main coronary artery to the left anterior descending artery. The red arrow depicts the left main coronary artery bifurcating to the left anterior descending artery.

**Figure 3:**
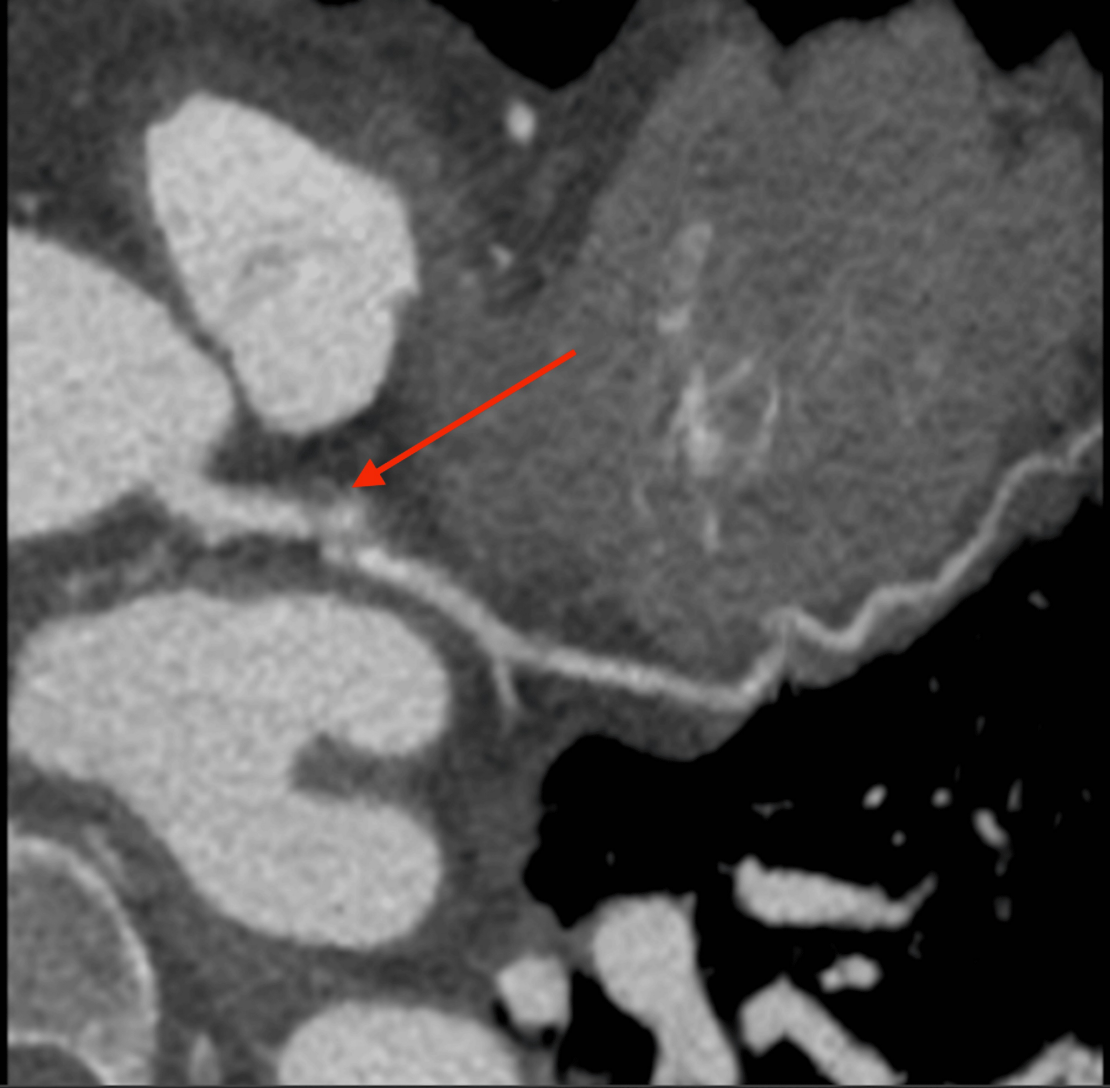
Computed tomographic angiogram of coronary arteries showed an 11 mm long by 9 mm wide nonthrombosed aneurysm emanating out of the distal end of the left main coronary artery to the proximal left circumflex. The red arrow depicts the left main coronary artery bifurcating to the proximal left circumflex.

Patient is a 61-year-old female with no prior history of coronary artery disease, heart failure, or valvular disease who presented with a new diagnosis of CAA. The patient initially presented with atypical chest pain. The chest pain was dull, non-radiating, and had no provoking factors. The presentation was not typical of acute coronary syndrome. She underwent a cardiovascular workup, including a stress test, which was negative for myocardial infarction or ischemia. CT angiogram of coronary arteries revealed a significant aneurysm of the coronary bifurcation into LAD and circumflex. This aneurysm has been monitored over several years with serial CT angiograms. The most recent CT angiogram is depicted in Figures 1-3.

**Figure 4:**
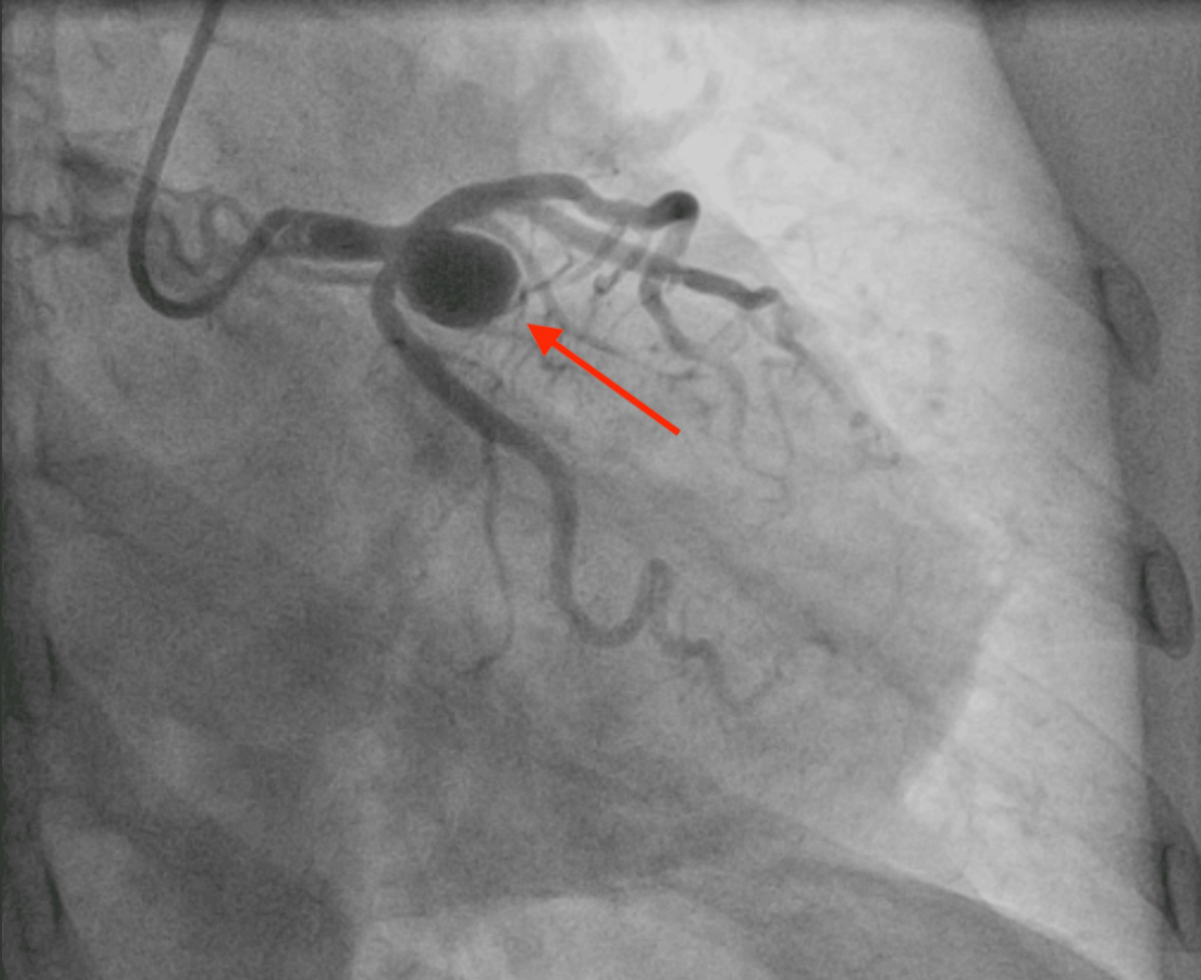
Left heart catheterization. The red arrow points to a giant aneurysm estimated to be around 16 mm originating from the distal L. main and involving the ostium of the left circumflex to a certain extent, the ostium of the left anterior descending artery.

Given the patient’s comorbidities, age, symptoms, and CT angiogram findings, the patient had an elective left heart catheterization (LHC). The LHC is shown in Figure 4. The discrepancy between LHC and CT angiogram was due to the LHC image being limited by its 2-dimensional vessel, whereas the CT coronary angiogram is 3-dimensional. This can result in different angles between the two modalities of the image. Furthermore, LHC is foreshortening, meaning the image can underestimate the vessel [5]. Given the growing coronary aneurysm, the decision was made to proceed to surgery. 

Treatment

The patient underwent repair of an aneurysm of the left main coronary artery. Intraoperative findings, the defect at the left main bifurcation likely compromised lumens of both LAD and circumflex; therefore, a left internal mammary artery graft was placed on a 0.2 cm LAD, and a vein graft was placed on a 0.2 cm obtuse marginal branch.

Outcome and follow-up

Postoperative transthoracic echo showed an ejection fraction of 60%. On postoperative day 2, the patient developed respiratory distress with hypoxia requiring initiation of BiPAP. Chest X-ray showed pulmonary edema and right pleural effusion. This is due to the patient developing transient junctional bradycardia. The patient was placed on an epicardial pacer and managed with intravenous diuretics combined with therapeutic thoracentesis, which significantly improved her clinical status. She was eventually weaned off to room air on postoperative day 5. The patient was discharged home on postoperative day 7 with a beta blocker and oral Lasix.

## Discussion

The Coronary Artery Surgery Study registry defines CAA as a dilation of more than 1.5 times the diameter of the normal adjacent artery. There is no universally accepted definition for what classifies as a GCAA. However, one criterion often referenced in the literature for what constitutes an aneurysm as “giant” is a vessel with a diameter at least four times greater than the reference vessel [6]. In our case, coronary angiography revealed a GCAA at the distal left main coronary artery measuring approximately 16 mm, which is more than four times the diameter of the reference vessel, estimated to be around 3.05 mm.

One of the common causes of CAA is atherosclerosis. Atherosclerosis accounts for approximately 50% of cases in adults, 20% to 30% are due to congenital causes, and 10% to 20% are due to inflammatory or connective tissue disorders such as Kawasaki disease. The histologic features of atherosclerosis consist of hyalinization, lipid deposition, disruption of intima and media, focal calcification, and fibrosis. In addition, calcification of the coronary artery accounts for 43% of the cases [4]. Studies suggest that a specific MMP gene variant (MMP3-5A) combined with atherosclerosis may increase the prevalence of CAA compared to atherosclerosis alone [4,7]. Future studies will need to address this hypothesis.

Coronary aneurysms have been found to be more common in men [7]. In angiographic studies, men represented 1.79% of the 4970 studies, and women represented 0.56% of the 2131 studies [7]. CAA is more common in men and in those with hyperlipidemia [8]. The right coronary artery is the most affected artery, which is around 40% compared to LAD (32%) and L. main being least affected (3.5%) [8]. In our case report, our patient is female, and the affected artery is the left main artery.

Coronary angiograms allow physicians to assess the structure of the coronary arteries. The gold standard for diagnosing CAA is coronary angiography, which evaluates the aneurysm anatomy [1]. Coronary angiography with intravascular ultrasound can help differentiate true aneurysm from false aneurysm, and provides a composition of the lumen and arterial wall structure [9]. Other non-invasive studies, including echocardiography and magnetic resonance imaging, can also be used to evaluate CAA.

Management of CAA has challenges due to its unknown etiology, as recommendations are based on anecdotal evidence or case series. Furthermore, there is a knowledge gap due to a lack of randomized trials or large-scale studies. Treatment varies based on clinical presentation, structure, and morphology. Many studies show surgery is favored due to fewer complications, such as thrombosis, rupture, and coronary embolization. Multiple surgical strategies implemented include reconstruction, resection, and isolation with coronary bypass [10]. Percutaneous coronary intervention (PCI) is also preferred in selected patients with 5mm to 10mm aneurysms [11]. However, in certain studies, patients with CAAs treated with PCI have shown higher rates of mortality, stent thrombosis, target vessel revascularization, and myocardial infarction during intermediate follow-up, particularly in complex or larger aneurysms [12]. There are also reports of the CAA recurrence after stent placement with an incidence rate of 0.2% to 2.3% [1]. While there is ongoing debate regarding the optimal medical therapy, one study reported 0% major adverse cardiac events (MACE) in patients with CAA treated with anticoagulation, compared to those not on anticoagulation [13,14]. However, more extensive randomized trials are needed to confirm these findings.

## Conclusions

In conclusion, GCAA remain a rare cardiovascular finding in the adult population. Given their low incidence and asymptomatic presentations, physicians face a challenge in diagnosing GCAA, resulting in incidental findings during imaging for unrelated conditions. The case of the 61-year-old female with a history of hypertension and hyperlipidemia underscores the importance of considering GCAA in the differential diagnosis when cardiovascular anomalies are detected, even in patients without classic symptoms. While atherosclerosis remains the common cause of GCAA in adult populations, the pathophysiology of GCAA remains unclear. This highlights a significant gap in current medical knowledge and underscores the need for further research to identify specific risk factors, optimize diagnostic strategies, and develop evidence-based management guidelines. As a result, misdiagnoses of GCAA can lead to potential complications, including rupture or thrombosis. Heightened clinical awareness and individualized treatment approaches are essential. This case contributes to the growing body of literature on GCAA and reinforces the necessity for continued vigilance and investigation in rare cardiovascular presentations.
